# Soil Quality Assessment in Tourism-Disturbed Subtropical Mountain Meadow Areas of Wugong Mountain, Central Southeast China

**DOI:** 10.3390/life12081136

**Published:** 2022-07-28

**Authors:** Sohel Rana, Ziheng Xu, Razia Sultana Jemim, Zhen Liu, Yanmei Wang, Xiaodong Geng, Qifei Cai, Jian Feng, Huina Zhou, Tao Zhang, Mingwan Li, Xiaomin Guo, Zhi Li

**Affiliations:** 1 College of Forestry, Henan Agricultural University, Zhengzhou 450002, China; sohel.sam@live.com (S.R.); liuzh20@163.com (Z.L.); wym3554710@163.com (Y.W.); xiaodonggeng@163.com (X.G.); caiqifei0416@163.com (Q.C.); 15713679647@163.com (J.F.); zhn100585@163.com (H.Z.); a17538392107@163.com (T.Z.); limingwan3@126.com (M.L.); 2 Palm Eco-Town Development Co., Ltd., Building 3A, Haihui Center, Zhengdong New Area, Zhengzhou 450000, China; xuzh1013@163.com; 3 College of Life Sciences, Henan Agricultural University, Zhengzhou 450002, China; jemim.sam@outlook.com; 4 College of Forestry, Jiangxi Agricultural University, Nanchang 330045, China; gxmjxau@163.com

**Keywords:** minimum data set, mountain meadow, soil quality index, Wugong Mountain, tourism disturbance

## Abstract

Meadow soil is a vital ecosystem component and can be influenced by meadow vegetation. Evaluating soil quality in mountain meadows subjected to different levels of tourism disturbance is essential for scientific research, ecological restoration, and sustainable management. This study aimed to evaluate meadow soil quality at different tourism-disturbance levels and attempted to establish a minimum data set (MDS) with compatible indicators for soil quality assessment of subtropical mountain meadows. We analyzed fifteen soil physical, chemical, and biological indicators in control check (CK), light disturbance (LD), medium disturbance (MD), and severe disturbance (SD) meadow areas in Wugong Mountain, west of Jiangxi, China. In addition, a soil quality index (SQI) was determined using the established MDS based on the integrated soil quality index. Average soil permeability, soil pH, available nitrogen (AN), available phosphorus (AP), and number of fungal OTUs were finally introduced into the MDS to evaluate meadow soil quality at different tourism-disturbance levels. The study found that the soil of the Wugong Mountain meadow was acidic, the bulk density was loose, and the nutrient content was rich. Additionally, SQI decreased with increase in tourism-disturbance level. The mean SQI values of the Wugong Mountain meadow areas were: CK, 0.612; LD, 0.493; MD, 0.448; and SD, 0.416. Our results demonstrate that the SQI based on the MDS method could be a valuable tool with which to indicate the soil quality of mountain meadow areas, and the SQI can be regarded as a primary indicator of ecological restoration and sustainable management.

## 1. Introduction

Soil is the basis for the survival of humans, animals, and plants [1], as well as the living space for numerous microorganisms [2]. Soil quality is the comprehensive expression of soil’s physical, chemical, biological, and other properties. If only analyzed from a single aspect, the differences in soil quality under the action of different environments or external factors cannot be effectively represented [3]. Domestic and foreign scholars have conducted studies on soil quality evaluation using different methods [4,5,6,7,8]. The comprehensive quality evaluation model method has been used to analyze the soil quality of an abandoned mine residue area, and it was considered that pH value, organic carbon, total phosphorus, calcium, and sulfur were the key factors [9]. Geographic information system (GIS) technology was used to analyze the soil quality of Sopron Town in Hungary and it was reckoned that heavy metal pollution was the key factor affecting the soil quality of the town; soil quality risk was determined for an urban park area, which laid a foundation for a follow-up study [3]. 

The soil quality of a *Phyllostachys heterocycle* (Carr.) Mitford cv. *Pubescens* forest, characterized by different woodland densities, was evaluated using the multi-index method and a reasonable density of Phyllostachys heterocycla (Carr.) Mitford cv. Pubescens forest was the key measure in controlling soil quality [10]. Currently, there is no unified method to evaluate the consistency of soil quality. Most calculation functions are determined according to the different index profiles of a given study area, and various scientific research evaluations are carried out according to different regional locations, environmental conditions, and evaluation purposes. The concept is to determine specific evaluation indicators and then conduct comprehensive screening, using different statistical methods for comprehensive calculation. A minimum data set (MDS) can reflect soil quality with a small number of indicators, which is useful for soil quality evaluation and detection [11] and has the advantage of reducing data redundancy and subjective human factors [12]. The MDS method was used to analyze 41 soil physicochemical and biological indicators of cold waterlogged paddy fields in Fujian Province and six factors were selected, including carbon and nitrogen ratio, bacteria, microbial biomass nitrogen, total reducible matter, physical sand, and total phosphorus, to form a MDS for use in analyzing soil quality in different regions [13]. In addition, soil quality was analyzed in Irish grassland under different management intensities; soil organic carbon, total nitrogen, soil particle density, bulk density, available potassium, and carbon-nitrogen ratio constituted the MDS for soil quality evaluation and it was determined that high-intensity artificial intervention would have adverse effects on the soil quality of the grassland [14].

The Wugong Mountain meadow in Jiangxi is a typical representative of subtropical mountain meadows. It has typicality and particularity in the vertical belt spectrum of vegetation in East China due to its vast area and low distribution datum altitude [15]. In recent years, mountain landscapes, such as southern mountain meadows, have been widely developed, with sharp increases in the numbers of tourists. The meadow ecosystem has been degraded to varying levels due to human trampling, and the grassland plants appear to be dwarf, poor, and sparse. Some even become bare surfaces, resulting in weakened ecosystem functions and reduced resilience [16,17]. In addition, with regard to the hyperspectral characteristics of several kinds of vegetation in this region, it was found that the spectral reflectance of vegetation exhibited the following order: *Carex chinensis* > *Arundinella anomala* > *Miscanthus sinensis* > *Sinarundinaria nitida* > *Fimbristylis wukunnshanensis* [15]. Studies have reported that under high-temperature conditions, the CO_2_ and N_2_O emission rates of *Miscanthus sinensis* soil in this area were lower than those of *Carex chinensis* and *Fimbristylis wukunnshanensis* [18,19]. Furthermore, the total amount of inorganic phosphorus in meadow soil was found to increase significantly with increasing elevation [20]. The content of alkali-hydrolyzable nitrogen in this region’s surface layer of meadow soil was greater than that in deep soil [21].

It should be noted that there are abundant mountain meadow resources in subtropical areas. While studying this type of ecosystem, previous scholars have focused on the development strategies and suggestions of the animal husbandry industry. In addition, southern meadows are characterized by poor palatability to livestock and are prone to ecological degradation due to thin turf. As a typical representative of the southern meadow ecosystem, Wugong Mountain has been the subject of relevant reports on the impact of tourism development, utilization, and flora research in recent years. However, the research on the particular mountain meadowland of Wugong Mountain has not attracted enough attention. We have made relevant analyses with regard to different aspects, such as vegetation characteristics, individual physical and chemical characteristics of soil, and tourism marketing strategies, but there is still a lack of more in-depth and systematic research. 

To our knowledge, systematic studies on the soil quality evaluation of subtropical mountain meadows have been less well documented. For this study, we selected as the scope of our research the core tourist areas, and fifteen soil physical, chemical, and biological indicators were determined. Our research attempted to establish an MDS with compatible indicators for soil quality assessment of subtropical mountain meadows. This study is a new attempt to demonstrate the variation in soil properties under different tourism-disturbance levels and verify the effectiveness of the MDS in this study area. The primary objectives of this study were: (1) to identify the variation in soil properties under three different tourism-disturbance levels, (2) to establish an MDS with the proper indicators for soil quality assessment, and (3) to evaluate the soil quality of different tourism-disturbance levels in the Wugong Mountain region using the SQI method and determine the controlling indicators in order to identify whether the MDS is useful for soil quality evaluation in meadow ecosystems and as a theoretical basis for practical applications related to sustainable ecological restoration and management.

## 2. Materials and Methods

### 2.1. Study Site

Wugong Mountain (114°10′–114°17′ E, 27°25′–27°35′ N) is at the junction of three administrative regions (Jian, Pingxiang, and Yichun City) of Jiangxi Province, China. It is the watershed of the Xiangjiang and Ganjiang river systems and stretches for about 120 km, with a total area of about 970 km^2^. The annual average temperature is 14–16 °C, and the highest temperature in summer is 23 °C. The average annual sunshine duration is 1580–1700 h, the average annual evaporation is 1360–1700 mm, the average annual humidity is 70–80%, and the average annual rainfall is 1350–1570 mm. Wugong Mountain rock types are mainly granite and gneiss, and the peak Baihefeng (Jinding) is about 1918.3 m above sea level [22]. Mountain meadows are distributed at an altitude of 1600 m to the top of the mountain range. The soil is subtropical mountain meadow soil, the vegetation mainly Miscanthus sinensis, Arundinella anomala, Perotis indica, etc., with a small number of Polygonaceae, Rosaceae, Labiatae, and Cruciferae plants. One of the most widespread species in the region is *Miscanthus sinensis* [23,24].

### 2.2. Experimental Design and Sample Collection

#### 2.2.1. Plot Setting

The Jinding (main peak) area of Wugong Mountain is one of the typical tourism-disturbance areas. In the meadow area, the vegetation grows well in the absence of tourists, and there is no other disturbance behavior except tourism activities. Therefore, tourism activities directly lead to the degradation of meadow vegetation coverage in the study area. In October 2019, the altitude (1900 m) range was selected under the condition of excluding differences in altitude, terrain, and other natural factors, with reference to the national standard (GB 19377—2003) of “grading index of natural grassland degradation, desertification and salinization” issued by the Administration of Quality Supervision, Inspection and Quarantine (AQSIQ) in 2004 [25] and the research results of relevant scholars on the grading standard of degraded grassland [26,27,28]. Based on tourism disturbance, the vegetation coverage rate (CR) decreases the relative percentage (%). A total of four samples were set up in this study; the samples were: control check (CK, CR ≥ 90%), light disturbance (LD, 60% ≤ CR < 90%), medium disturbance (MD, 30% ≤ CR < 60%), and severe disturbance (SD, CR < 30%). The three 10 m × 10 m repeated plots were randomly set for each sample to assess the soil quality of mountain meadows with different disturbance levels. A basic overview of the different research treatments and sample plots of mountain meadows is shown in Table 1.

#### 2.2.2. Sample Collection and Determination

(1) The methods for the collection and determination of soil chemical properties, soil enzymes, and microorganisms

In each 10 m × 10 m quadrat, five sampling points were carried out along two diagonal lines and their intersection points. The samples were collected from each point at a soil depth of 0–20 cm, and the samples were mixed. About 500 g of soil was removed by the quartering method (a 100 g soil sample from each sampling point) and put into fresh-keeping bags. Two circular knives were used for sampling (the circular knives were stainless, the upper and lower covers were aluminum, the specification was 50.46 mm × 50 mm, and the cubage was 100 cm^3^). The study was conducted according to the standard list of experiments and calculation methods [29,30]. The soil samples were returned to the laboratory for natural air-drying, and plants, animal residues, and stones were removed. The soil was carefully crushed, and samples were prepared for chemical indicator and soil enzyme analysis. Soil pH, organic matter (OM), total nitrogen (TN), total phosphorus (TP), total potassium (TK), available nitrogen (AN), available phosphorus (AP), available potassium (AK), and other chemical indicators were determined by conventional analysis methods [31]. Soil enzymes were determined by the Guansongmeng method [32], sucrase by invertase 3,5-Dinitrosalicylic acid colorimetry, soil catalase by the volumetric method, and urease by indophenol blue colorimetry. In addition, about 50 g of soil was taken, and the samples were immediately put into a dry ice low-temperature box. Afterward, the samples were entrusted to the Beijing Nohe Zhiyuan Biological Information Technology Co., Ltd. for high-throughput sequencing of microbial diversity. Soil bacteria were analyzed using 16S rDNA amplicon sequencing technology, with the V3 and V4 areas selected for amplification, and in fungal 18S rDNA sequences were analyzed. The sample attribution was first determined at higher levels, followed by lower-level attribution analysis based on ITS1 sequences. Bacteria and fungi were all sequenced on a Illumina HiSeq2500 sequencing platform using the paired-end sequencing (paired-end) method to construct small fragment libraries for double-end sequencing, filtered by splicing on reads, OTU (operational taxonomic units) clustering, and, later, species and diversity analysis [33]. Since some OTU results could not be annotated when species interpretation was conducted (to avoid information loss), the diversities of bacteria and fungi were represented by their respective OTU numbers.

(2) Methods for the collection of soil samples and the determination of soil physical properties

In each 10 m × 10 m sample plot, three sampling points were selected according to the shape of the “pin” or along the diagonal line. The spacing of each point was about 5 m. Sampling was conducted with two ring knives in a 0–20 cm soil layer (the ring cutter body was made of stainless steel, and the upper and lower covers were made of aluminum). The specification was 50.46 mm (diameter) × 50 mm (height), and the volume was 100 cm^3^. This was in accordance with the experimental operation and calculation methods listed in the forestry industry standards of the People’s Republic of China, “Determination of forest soil water-physical properties [30]” and “Determination of forest soil percolation rate [29]”, combined with the research results of relevant scholars [34]. Drying and infiltration methods were used to measure sample volume weight and average infiltration rate. The following formula was used:

$$\mathrm{The}\;\mathrm{average}\;\mathrm{infiltration}\;\mathrm{rate}=\frac{\mathrm{The}\;\mathrm{total}\;\mathrm{amount}\;\mathrm{of}\;\mathrm{seepage}\;\mathrm{at}\;\mathrm{the}\;\mathrm{time}\;\mathrm{of}\;\mathrm{steady}\;\mathrm{infiltration}}{\mathrm{The}\;\mathrm{time}\;\mathrm{when}\;\mathrm{the}\;\mathrm{steady}\;\mathrm{infiltration}\;\mathrm{reached}}$$

Since the permeability rate of all soil samples reached a stable level before 60 min, for the convenience of comparisons, the total amount of infiltration was the same as that in the previous 60 min.

### 2.3. Calculation Method for the Soil Quality Comprehensive Index

#### 2.3.1. Collation of Basic Data Sets

The results of the fifteen indicators in meadow soil were summarized using Microsoft Excel v. 2016 (Microsoft Corp., Redmond, WA, USA). In addition, the basic data set for soil quality evaluation, the SPSS v. 26 (IBM Corp., Armonk, NY, USA) program used for descriptive statistics, principal component analysis, and the functional model were used to determine the overall soil quality.

#### 2.3.2. Construction of the Minimum Data Set

The SPSS program was used to analyze the principal components of fifteen indicators in the basic data set and calculate the principal components whose characteristic roots were greater than 1. The indicators with a principal component factor load greater than or equal to 0.5 in each column were divided into groups. If an indicator load was greater than or equal to 0.5 in two groups of principal components, the index was merged into the group with a lower correlation with other indicators. We calculated each group’s norm value, selected the index whose norm value was less than 10% of the highest score, and analyzed the correlation of the selected indicators in each group. If a high correlation (r > 0.5) was found, the index with a high score was determined to enter the MDS to obtain the final MDS. The norm value represents the ability to interpret comprehensive information, and the calculation formula used was as follows:

(1) $$N_{ik}=\sqrt{\overset{k}{\underset{i=1}{\sum }}\left(u_{ik}^{2}\lambda_{k}\right)}$$

In the formula, Nik is the comprehensive load of the *i*-th variable on the first *k* principal components whose eigenvalue is greater than 1; *u_ik_* is the load of the *i*-th variable on the *k*-th principal component; and *λ_k_* is the characteristic root of the *k*-th principal component.

#### 2.3.3. Comprehensive Evaluation Index of Soil Quality

The formula used for the soil quality comprehensive evaluation index was as follows [10,11]:

(2) $$SQI=\overset{n}{\underset{i=1}{\sum }}W_{i}\times N_{i}$$

In the formula, the soil quality index (*SQI*) is the comprehensive evaluation index of soil quality; *W_i_* is the index weight coefficient; and the Person correlation analysis in SPSS 21.0 was used to calculate the correlation coefficient of each index. The ratio of the average value of the correlation coefficient between an indicator and other indicators to the average value of the correlation coefficient of all evaluation indicators is the weight coefficient of the index; *N_i_* is the membership degree, and *n* is the number of indicators.

Since changes in soil indicators are continuous, the continuous membership function was used to standardize the indicators, and the ascending and descending properties of the membership functions were determined by using the positive and negative characteristics of the load of the principal component factors.

The formula of the “S” ascending membership function is:

(3) $$F\left(X\right)=\{\begin{array}{cc} 1 & \left(X\geq X_{max}\right)\\ 0.9\times \frac{X-X_{min}}{X_{max}-X_{min}}+0.1 & \left(X_{max}\geq X\geq X_{min}\right)\\ 0.1 & \left(X\leq X_{min}\right) \end{array}$$

The formula of the “S” descending membership function is:

(4) $$F\left(X\right)=\{\begin{array}{cc} 1 & \left(X\geq X_{max}\right)\\ 0.9\times \frac{X_{max}-X}{X_{max}-X_{min}}+0.1 & \left(X_{max}\geq X\geq X_{min}\right)\\ 0.1 & \left(X\leq X_{min}\right) \end{array}$$

In the formula, X*_min_* and X*_max_* are the minimum and maximum values of soil evaluation indicators.

## 3. Results

### 3.1. Descriptive Statistics of Soil Physicochemical Properties, Microorganisms, and Enzyme Activities in the Mountain Meadow

**Figure 1:**
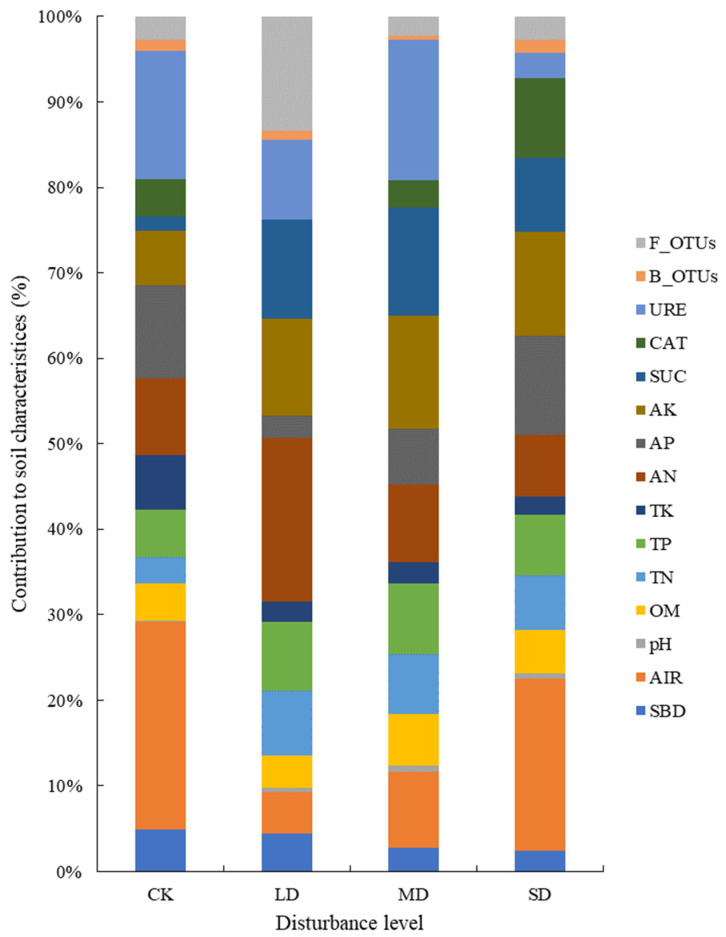
The individual contributions of meadow soil properties in areas of different tourism-disturbance levels. Abbreviations: SBD, soil bulk density; AIR, average infiltration rate; pH, soil pH; OR, organic matter; TN, total nitrogen; TP, total phosphorus; TK, total potassium; AN, available nitrogen; AP, available phosphorus; AK, available potassium; SUC, sucrase; CAT, catalase; URE, urease; B_OTUs, bacterial OTU number; F_OUTs, fungal OTU number.

The physical properties of mountain meadow soil with different tourism-disturbance levels in the Wugong Mountain region are shown in the descriptive statistical results presented in Table 2. The mean volume of the bulk density increased with the disturbance level, while the average permeability shows that the disturbance meadow area was reduced compared to CK. Regarding chemical properties, the mean value of soil pH decreased with the increase in disturbance level. The mean values of organic matter, total nitrogen, total phosphorus, and available nitrogen were slightly higher than CK in the disturbance area. The total potassium levels in the MD and SD regions were lower than the corresponding CK and LD levels. Available phosphorus in the disturbance areas increased with the increase in disturbance level, but the average values for the LD and MD regions were lower than the value for CK. Available potassium decreased with the increase in disturbance level, but the LD area value was slightly higher than that of the CK area. The individual contributions of the various meadow soil properties in areas of different tourism-disturbance levels are shown in Figure 1.

Regarding soil biological characteristics, the activities of soil invertase in the disturbance areas were significantly lower than in the CK area. The catalase activities in the LD and MD areas were equal to that in the CK area, but the activity was lower in the SD region. Soil urease activity in the disturbance areas was significantly lower than in the CK region. The number of soil bacterial OTUs decreased with the disturbance level, but in the SD, the value was increased. The number of soil fungal OTUs increased with the disturbance levels. The coefficients of variation for each index showed weak variation (CV ≤ 10%) or moderate variation (10% < CV ≤ 100%). On the whole, the soil in the study area was acidic and bulk density was loose, while nutrient contents and bacterial and fungal presence were relatively rich. The effect of different tourism-disturbance levels on the soil properties was different. It was necessary to take the data for each index as a basis for comprehensively evaluating the quality of meadow soil in different disturbance areas.

### 3.2. Determination of the MDS for Soil Quality Evaluation of Mountain Meadows

The results of the principal component analysis showed that the eigenvalues of the first five principal components were 5.809, 2.474, 2.025, 1.474, and 1.117, respectively (Table 3). The variance contribution rates were 38.728%, 16,491%, 13,499%, 9.825%, and 7.446%. The total cumulative contribution rate was 85,989%, while the cumulative contribution rate of the first four principal components reached 78,542%, which is greater than 70% and meets the requirements for explaining system variation information. 

According to the load data for the principal component factors, the factors with an absolute value greater than 0.5 were selected and grouped. The indicators entered into the first group were bulk density (A1), pH (A3), organic matter (A4), total nitrogen (A5), total phosphorus (A6), sucrase (A11), and catalase (A12). The indicators entered into the second group were total potassium (A7), available potassium (A10), urease (A13), and bacterial OTU number (A14). The third group’s indicators were average infiltration rate (A2) and available nitrogen (A8). Finally, the fourth group was the number of fungal OTUs (A15). Since, for the first four principal components, the load of each factor belonging to the group was greater than the value of the fifth principal component, the basic data set was divided into four groups.

Combining the feature of each indicator factor loading and characteristic root, the norm value for each variable was calculated. It can be seen that the highest norm value for the first group was 2.266 (A3), while that for the second group was 1.84 (A9), that for the third group was 1.616 (A8), and that for the fourth group was 1.268 (A15). In each group, the indicator which was less than 10% of the highest norm value of the group was taken and combined with the correlation indicator (Table 4). If the absolute value of the correlation coefficient between the indicators in the same group was greater than 0.5, the indicator with the higher norm value was retained. Finally, five indicators, such as average soil permeability (A2), pH (A3), available nitrogen (A8), available phosphorus (A9), and fungal OTU quantity (A15), were determined to be entered in the MDS for soil quality evaluation of mountain meadows.

### 3.3. The Comprehensive Evaluation of the Soil Quality of Mountain Meadows at Different Tourism-Disturbance Levels

The soil in the study area was mountainous meadow soil; good soil permeability represents a better water conservation function. The data analysis results showed that soil pH and average permeability are the core factors in the soil quality evaluation of mountain meadows. While the soil was generally acidic, the increase in pH indicated a benign trend in soil quality. The amounts of available nitrogen, available phosphorus, and fungal OTUs in the soil were all positive indicators of soil fertility. Therefore, the membership value for mountain meadow soil quality evaluation had an “S” ascending function, and, according to the correlation coefficients between each indicator in the MDS, the weight coefficient was calculated by referring to the following method (Table 5). From the data analysis results, it can be seen that soil pH and average permeability are the core factors in the soil quality evaluation of mountain meadows.

**Figure 2:**
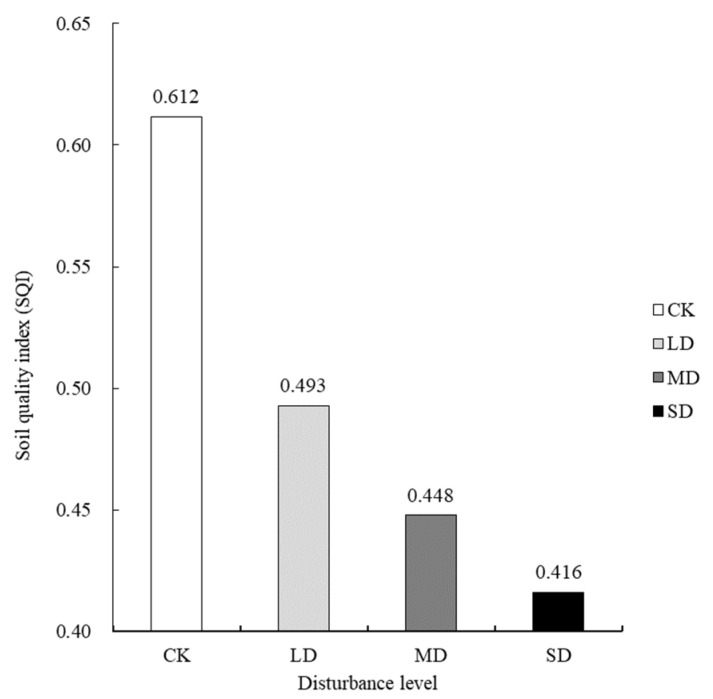
Comprehensive soil quality indexes of mountain meadow areas with different tourism-disturbance levels. Abbreviations: CK, control check; LD, light disturbance; MD, medium disturbance; SD, severe disturbance.

According to the membership value and weight coefficient of the MDS index for soil quality evaluation, the soil quality indexes of mountain meadows subjected to different levels of tourism disturbance were calculated according to Formula (2) (Figure 2). The results showed that the ranking of soil quality for mountain meadow areas subjected to different levels of tourism disturbance was CK > LD > MD > SD, and the soil quality indexes were 0.612, 0.493, 0.448, and 0.416, respectively, indicating that soil quality decreased with the increase in disturbance level and that only the soil quality of the CK area was in the middle-to-high level. The soil quality at each disturbance level decreased; the quality index of LD was 19.45%, that of MD was 26.80%, and that of SD was 32.00%—all lower than that of CK.

## 4. Discussion

### 4.1. Soil Quality Evaluation of Mountain Meadows

This study considered fifteen indicators of soil physical, chemical, and biological properties in typical subtropical mountain meadow areas of Wugong Mountain. On the basis of mathematical statistics and analysis, five indicators (average soil permeability, pH, available nitrogen, available phosphorus, and fungal OTUs) were selected for a minimum data set (MDS) to obtain a comprehensive index of meadow soil quality given different tourism-disturbance levels in the Wugong Mountain region. The results showed that the meadow soil in the study area was acidic, that bulk density was loose, and that the soil organic matter, total nitrogen, available nitrogen, and other nutrient contents and microbial presences were rich. If the comprehensive index of soil quality is greater than 0.5, this indicates that the soil quality is good [35]. The SQI of the meadow in the study area without tourism disturbance was greater than 0.5, while it was less than 0.5 in the tourism-disturbed areas. The comprehensive index of soil quality decreased with the increase in disturbance levels. As a result, the soil quality of meadows in tourism-disturbed areas is worse than in meadows without tourism disturbance. 

Studies have reported that comprehensive soil quality was significantly decreased with increase in tourism disturbance [36,37], which was consistent with the results of this study. The mountain meadow was rich in terms of the root system and there was a large amount of humus in the soil. Due to the low temperature, slow microbial decomposition, and high organic matter content, the soil bulk density was loose, but the nutrient content was rich [38]. The source of soil nutrients is mainly the return of nutrients from surface vegetation and underground roots. A previous study [20] has shown that the distribution of soil nutrients in Wugong Mountain meadowland shows strong surface aggregation. 

However, the disturbance behavior of tourists has reduced the soil surface vegetation of mountain meadows in Wugong Mountain, affected the source of soil nutrient return, and destroyed the soil structure, which has had a negative impact on soil bulk density, porosity, and permeability. The changes in soil quality with different disturbance levels are comprehensively reflected through the five indicators included in the MDS. In disturbed areas with low comprehensive indexes of soil quality, appropriate methods should be selected for vegetation restoration to prevent further degradation of soil quality, which results in the loss of the survival basis of vegetation and the deterioration of regional ecologies.

### 4.2. Soil Quality Evaluation Method Based on the MDS and the SQI Model

The combination of the MDS and the SQI can enable the effective evaluation of soil quality under different environmental or external factors. However, there is no unified standard for the determination of a minimum data set [35], including the membership function and weight value in the process of calculating the comprehensive evaluation index of soil quality, and there is also a lack of a unified calculation process [10]. In practice, the calculation function is usually determined according to different indicator profiles of the study area. However, the calculation function has been based on different calculation methods for soil quality evaluation [39,40], such as fuzzy mathematics, artificial neural networks, grey system theory, principal component analysis, etc. Different evaluations of the soil quality of a certain region may obtain different data, but the overall results should be similar [12,35]. 

In the Wugong Mountain meadow distribution area, previous studies have analyzed different characteristics of or indicators in the soil [41,42], and conclusions have also been based on certain aspects of research [43]. There is a lack of a systematic and representative evaluation metric, but the minimum data set (MDS) can be used to reflect soil quality statistically, ensuring a more accurate evaluation of soil quality [12].

## 5. Conclusions

The meadow soil in Wugong Mountain was found to be acidic, loose in terms of bulk density, and rich in nutrients. Five indicators, including average soil permeability, pH, available nitrogen, available phosphorus, and number of fungal OTUs, can be used as a minimum data set (MDS) to obtain a comprehensive index of soil quality for areas subjected to different levels of tourism disturbance in the Wugong Mountain region. Among the indicators, soil average permeability and pH are the core factors in soil quality evaluation. Comprehensive soil quality indexes decreased with increase in tourism-disturbance level. The soil quality index ranking with respect to different tourism-disturbance levels was CK > LD > MD > SD, and the soil quality indexes were 0.612, 0.493, 0.448, and 0.416, respectively. Based on the relevant experimental basis and data indicators, this study analyzed the impact of tourism disturbance on the soil of Wugong Mountain meadowland and made an objective evaluation. At present, though limited in terms of timescale and research scope, the research results can be used as an essential reference for short-term scientific research and productive work. A long-term study with a more extensive range and including more indicators is required to further optimize and improve the evaluation method and system for the analysis of subtropical mountain meadow soils.

## Figures and Tables

**Table 1:** The geographic positions of the mountain meadow areas with different treatments.

Experimental Treatments	Elevation (m)	Slope Degree (°)	Slope Direction	Longitude (E)	Latitude (N)	Main Vegetation Type	Vegetation Coverage Rate (%)
CK	1907	7	NE25°	114°10′26.09	27°27′16.76	*Miscanthus* *sinensis*	98
	1904	9	NE27°	114°10′25.16	27°27′20.22	*Miscanthus* *sinensis*	97
	1903	6	NE29°	114°10′24.79	27°27′20.60	*Miscanthus* *sinensis*	100
LD	1914	5	NE23°	114°10′24.18	27°27′16.22	*Miscanthus* *sinensis*	76
	1917	8	NE27°	114°10′23.29	27°27′19.03	*Miscanthus* *sinensis*	82
	1901	7	NE26°	114°10′24.36	27°27′20.92	*Miscanthus* *sinensis*	73
MD	1912	6	NE24°	114°10′25.24	27°27′16.24	*Miscanthus* *sinensis*	47
	1910	8	NE28°	114°10′23.59	27°27′16.74	*Miscanthus* *sinensis*	55
	1911	<5	NE25°	114°10′23.41	27°27′17.16	*Miscanthus* *sinensis*	39
SD	1912	7	NE20°	114°10′24.87	27°27′16.18	*Miscanthus* *sinensis*	21
	1918	6	NE23°	114°10′23.04	27°27′17.00	*Miscanthus* *sinensis*	15
	1917	<5	NE25°	114°10′21.79	27°27′14.76	*Miscanthus* *sinensis*	19

CK: control check (no disturbance); LD: light disturbance; MD: medium disturbance; SD: severe disturbance; NE: north of due east.

**Table 2:** Descriptive statistics for meadow soil characteristics in areas with different tourism-disturbance levels.

Index	CK	LD	MD	SD
Soil bulk density (g·cm^−3^)	0.6 ± 0.03 ^a^	0.81 ± 0.02 ^b^	0.83 ± 0.05 ^b^	0.89 ± 0.03 ^b^
Average infiltration rate (mm·min^−1^)	10.64 ± 8.46 ^a^	4 ± 0.43 ^ab^	4.39 ± 1.04 ^ab^	2.25 ± 1.59 ^b^
Soil pH	4.74 ± 0.01 ^a^	4.67 ± 0.05 ^a^	4.54 ± 0.1 ^a^	4.47 ± 0.1 ^b^
Organic matter (g·kg^−1^)	90.52 ± 13.15 ^ab^	87.96 ± 7.58 ^abc^	119.03 ± 19.26 ^bc^	115.16 ± 20.13 ^c^
Total N (g·kg^−1^)	3.84 ± 0.22 ^ab^	4.24 ± 0.41 ^abc^	5.18 ± 0.56 ^c^	4.9 ± 0.64 ^bc^
Total P (g·kg^−1^)	1.02 ± 0.11 ^ab^	1.01 ± 0.1 ^ab^	1.4 ± 0.18 ^ab^	1.53 ± 0.22 ^a^
Total K (g·kg^−1^)	47.67 ± 5.85 ^a^	48.25 ± 1.5 ^a^	38.75 ± 1.53 ^ab^	41.42 ± 1.8 ^ab^
Available nitrogen (mg·kg^−1^)	282.24 ± 82.13 ^abc^	337.92 ± 144.18 ^ab^	382.02 ± 93.13 ^a^	295.47 ± 74.19 ^abc^
Available phosphorus (mg·kg^−1^)	27.42 ± 9.84 ^ab^	19.71 ± 1.18 ^b^	25.59 ± 4.46 ^ab^	39.23 ± 15.82 ^c^
Available potassium (mg·kg^−1^)	82.67 ± 17.13 ^a^	89.95 ± 22.68 ^a^	76.06 ± 27.09 ^a^	70.2 ± 29.91 ^a^
Sucrase (mg·g^−1^)	80.42 ± 4.45 ^a^	59.74 ± 15.43 ^a^	67.47 ± 22.72 ^a^	54.8 ± 16.54 ^a^
Catalase (mg·g^−1^)	0.07 ± 0.01 ^b^	0.07 ± 00.01 ^b^	0.07 ± 0.01 ^b^	0.05 ± 0.02 ^a^
Urease (mg·g^−1^)	0.55 ± 0.27 ^a^	0.37 ± 0.08 ^a^	0.37 ± 0.16 ^a^	0.41 ± 0.04 ^a^
Bacterial OTU number	1635 ± 73 ^a^	1620 ± 37 ^a^	1544 ± 21 ^a^	1722 ± 93 ^b^
Fungal OTU number	1112 ± 97 ^a^	1177 ± 352 ^a^	1174 ± 71 ^a^	1252 ± 118 ^a^

Notes: CK: control check (no disturbance); LD: light disturbance; MD: medium disturbance; SD: severe disturbance. Data are mean values ± SE. Different lowercase letters with in the same raw for each index indicate a significance differences at *p* < 0.05, respectively.

**Table 3:** Calculation results for the principal component load matrix and norm values.

Index	Principal Component Load Matrix					Grouping	Norm Value
	PC1	PC2	PC3	PC4	PC5		
A1	0.652	−0.227	−0.135	0.442	0.345	1	1.749
A3	−0.932	−0.051	0.136	−0.031	−0.200	1	2.266
A4	0.803	0.134	0.247	−0.426	0.229	1	2.060
A5	0.882	−0.074	0.416	−0.107	0.026	1	2.215
A6	0.874	0.128	0.133	−0.109	0.067	1	2.131
A11	−0.682	0.075	−0.307	−0.152	0.340	1	1.751
A12	−0.731	−0.168	0.286	0.076	0.445	1	1.889
A7	−0.482	0.523	0.388	0.304	−0.333	2	1.610
A10	−0.160	−0.587	0.400	0.112	−0.490	2	1.269
A13	−0.335	0.775	0.333	0.148	0.127	2	1.553
A14	0.264	0.747	−0.237	0.418	−0.017	2	1.469
A9	0.620	0.627	−0.110	−0.171	−0.315	2	1.840
A2	−0.493	0.318	0.587	−0.093	0.330	3	1.580
A8	0.417	−0.194	0.843	0.216	0.043	3	1.616
A15	0.295	−0.192	−0.122	0.811	0.097	4	1.268
Characteristic root	5.809	2.474	2.025	1.474	1.117		
Variance contribution rate (%)	38.728	16.491	13.499	9.825	7.446		
Cumulative contribution rate (%)	38.728	55.218	68.717	78.542	85.989		

**Table 4:** Correlation coefficient matrix of soil indicators.

Index	A1	A2	A3	A4	A5	A6	A7	A8	A9	A10	A11	A12	A13	A14	A15
A1	1.00														
A2	−0.45	1.00													
A3	−0.67 *	0.49	1.00												
A4	0.37	−0.08	−0.72 **	1.00											
A5	0.47	−0.22	−0.76 **	0.83 **	1.00										
A6	0.47	−0.22	−0.84 **	0.79 **	0.84 **	1.00									
A7	−0.34	0.39	0.51	−0.45	−0.33	−0.36	1.00								
A8	0.30	0.17	−0.29	0.42	0.73 **	0.40	0.09	1.00							
A9	0.04	−0.28	−0.58 *	0.53	0.49	0.70 *	0.04	0.01	1.00						
A10	−0.19	0.07	0.31	−0.26	0.03	−0.03	0.07	0.33	−0.31	1.00					
A11	−0.44	0.34	0.51	−0.49	−0.68 *	−0.37	0.08	−0.60 *	−0.28	−0.05	1.00				
A12	−0.30	0.53	0.56	−0.52	−0.48	−0.54	0.33	0.02	−0.69 *	0.14	0.60 *	1.00			
A13	−0.34	0.57	0.31	−0.07	−0.25	−0.22	0.67 *	0.04	0.17	−0.35	0.18	0.21	1.00		
A14	0.20	0.04	−0.34	0.05	0.03	0.31	0.28	−0.18	0.58	−0.44	−0.12	−0.33	0.36	1.00	
A15	0.49	−0.26	−0.31	−0.12	0.15	0.19	−0.21	0.23	−0.03	0.14	−0.14	−0.17	−0.09	0.27	1.00

Note: * and ** represent significance differences at *p* < 0.05 and *p* < 0.01, respectively.

**Table 5:** Weight coefficient of soil quality evaluation index.

Index	Weight Coefficient	Subordinate Function
A2	0.228	$F\left(X\right)=\{\begin{array}{cc} 1 & \left(X\geq X_{20.35}\right)\\ 0.9\times \frac{X-X_{0.99}}{X_{20.35}-X_{0.99}}+0.1 & \left(X_{20.35}\geq X\geq X_{0.99}\right)\\ 0.1 & \left(X\leq X_{0.99}\right) \end{array}$
A3	0.317	$F\left(X\right)=\{\begin{array}{cc} 1 & \left(X\geq X_{4.75}\right)\\ 0.9\times \frac{X-X_{4.36}}{X_{4.75}-X_{4.36}}+0.1 & \left(X_{max}\geq X\geq X_{4.36}\right)\\ 0.1 & \left(X\leq X_{4.36}\right) \end{array}$
A8	0.132	$F\left(X\right)=\{\begin{array}{cc} 1 & \left(X\geq X_{504.39}\right)\\ 0.9\times \frac{X-X_{233.18}}{X_{504.39}-X_{233.18}}+0.1 & \left(X_{max}\geq X\geq X_{233.18}\right)\\ 0.1 & \left(X\leq X_{233.18}\right) \end{array}$
A9	0.167	$F\left(X\right)=\{\begin{array}{cc} 1 & \left(X\geq X_{50.85}\right)\\ 0.9\times \frac{X-X_{18.39}}{X_{50.85}-X_{18.39}}+0.1 & \left(X_{max}\geq X\geq X_{18.39}\right)\\ 0.1 & \left(X\leq X_{18.39}\right) \end{array}$
A15	0.156	$F\left(X\right)=\{\begin{array}{cc} 1 & \left(X\geq X_{1441}\right)\\ 0.9\times \frac{X-X_{777}}{X_{1441}-X_{777}}+0.1 & \left(X_{max}\geq X\geq X_{777}\right)\\ 0.1 & \left(X\leq X_{777}\right) \end{array}$

## Data Availability

The data sets generated during and/or analyzed during the current study are available from the corresponding author upon reasonable request.
